# Evaluating the Effectiveness of the Chemical-Mass Casualty Incident Response Education Module (C-MCIREM): A Pilot Simulation Study With a Before and After Design

**DOI:** 10.7759/cureus.17980

**Published:** 2021-09-14

**Authors:** Myeong-sik Kim, Heejun Shin, Giwoon Kim, Jae Hyuk Kim, Sori Kang, Tai Been Kang, Jeong Gyun Kim

**Affiliations:** 1 Emergency Medicine, Soonchunhyang University Hospital, Bucheon city, KOR; 2 Emergency Medicine, Soonchunhyang University Hospital, Bucheon, KOR; 3 Emergency Medicine, Mokpo Hankook Hospital, Mokpo, KOR; 4 Emergency Medicine, National Emergency Medical Center, Seoul, KOR

**Keywords:** mass casualty incident, simulation in medical education, chemical incident, disaster preparedness and response, emergency medicine - emergency critical care - disaster medicine

## Abstract

Background

With the occurrence of a number of major disasters around the world, there is growing interest in chemical disaster medicine. In South Korea, there is a training program for mass casualty incidents (MCI) and backup by legal regulations by the Framework Act on the Management of Disasters and Safety. However, there is no program focusing on chemical disasters. Thus, the authors newly created a program, the Chemical-Mass Casualty Incident Response Education Module (C-MCIREM) in September 2019. This was a pilot study to verify the educational effect of the program.

Method

A pre/post study was conducted of a chemical MCI training program based on simulation. A total of 25 representative and qualified participants were recruited from fire departments, administrative staff of public health centers, and healthcare workers of hospitals in the Gyeonggi-do province of South Korea. They participated in a one-day training program. A knowledge test and confidence survey were provided to participants just before training, and again immediately following the training online. The authors compared improvements of pre/post-test results. In the tabletop drill exercise, quantified qualitative analyses were used to measure the educational effect on the participants.

Results

In the knowledge test, the mean (standard deviation) scores for all 25 participants at baseline and after training were 41.72 (15.186) and 77.96 (11.227), respectively (*p* < 0.001). In the confidence survey for chemical MCI response for all 25 participants, all the sub-items concerning personal protective equipment selection, antidote selection, antidote stockpiling and passing on knowledge to colleagues, zone setup and decontamination, and chemical triage were improved compared to the baseline score (*p* < 0.001). The tabletop exercise represented a prehospital setting and had 11 participants. The self-efficacy qualitative survey showed pre- and post-exercise scores of 64/100 and 84/100 respectively. For a hospital setting exercise, it had 14 participants. The survey showed pre/post-exercise scores of 26/100 and 73/100 respectively. Twenty-two (88%) participants responded to the final satisfaction survey, and their overall mean scores regarding willingness to recommend this training program to others, overall satisfaction with theoretical education, overall satisfaction with tabletop drill simulation, and opinion about whether policymakers need this training were all over 8 out of 10 respectively.

Conclusion

C-MCIREM, the newly created chemical MCI program, provided effective education to the selected 25 participants among Korean chemical MCI responders in terms of both knowledge and practice at a single pilot trial. Participants were highly satisfied with the educational material and their confidence in disaster preparedness was clearly improved. In order to prove the universal educational effect of this C-MCIREM in the future, more education is needed.

## Introduction

With the occurrence of a number of major disasters around the world over the past 40 years, including the COVID-19 pandemic, there is a growing need for disaster training [1-12]. Accordingly, various disaster medicine educational programs have been developed and their effectiveness has been tested [1-12]. In particular, chemical disasters, such as the Seveso dioxin leakage disaster in Italy, the methyl isocyanate leakage disaster in Bhopal, India, and the Gumi fluoric acid gas leakage disaster in Korea in 2012, have attracted a great deal of attention worldwide [13-16]. Toxic chemicals continue to cause environmental problems, such as air, soil, and groundwater pollution, and result in secondary damage with long-term exposure due to their differences in relation to mass casualty incidents (MCIs) in which trauma is the main cause of damage [13-16].

In South Korea, in accordance with the Framework Act on the Management of Disasters and Safety enacted in 2016, the Emergency Response Manual in Disaster Fields specifies that a Disaster Medical Assistance Team (DMAT) consisting of Emergency Medical Services (EMS) personnel from the fire department, rapid response teams from public health centers, and medical staff from emergency medical centers of regional hospitals should be dispatched to provide medical care in the event of a disaster or MCI [17, 18]. To prepare for such situations, the National Emergency Medical Center (NEMC) under the Ministry of Health and Welfare developed a national disaster medical training program named Korean Disaster Life Support (KDLS)-Field and has been conducting annual education for DMAT since 2016 [17, 18]. Furthermore, KDLS-Hospital training for hospital medical and administrative personnel has been developed and was first implemented in 2018 to resolve problems of surge capacity in hospitals when large numbers of patients at sites of disasters flood the hospital emergency department [17]. However, these two KDLS training programs mainly target trauma-based MCIs, and a KDLS-based response training program focusing on chemical disasters has not been developed.

In the existing KDLS-Field education, it is difficult to classify patients appropriately in special situations, such as chemical disasters, because the Move, Assess, Sort, Send (MASS) triage for the first stage and the Simple Triage and Rapid Treatment (START) method for the second stage were developed, focusing on the classification of trauma patients in disaster triage in the field. Therefore, education specific to chemical MCIs was needed, and it was necessary to take into consideration the concepts of zone setup, chemical triage, decontamination, personal protective equipment (PPE), and antidote in disaster management, given the characteristics of chemical disasters [19-23]. In Gyeonggi-do, South Korea, the development of regional-based chemical disaster education and pilot training began in 2019 to provide support through disaster medical management personnel dispatched from NEMC and medical staff selected by the Emergency Medical Association consisting of seven regional hospitals within the jurisdiction [17, 18, 24].

The authors developed the one-day course of chemical disaster education program based on the tabletop map drill exercise, named the Chemical-Mass Casualty Incident Response Education Module (C-MCIREM).

This study was performed to determine the educational effect of this program by evaluating the effectiveness of and self-confidence in improving the disaster response capacity before and after education in individuals and teamwork after training for preparing and responding to chemical disasters using C-MCIREM for first responders, health care providers, and public health center administrators.

## Materials and methods

Development of C-MCIREM

Gyeonggi-do has a population of over 13 million in 2019 or about a quarter of the population of South Korea [24]. Medical staff selected by the Emergency Medical Association from seven regional hospitals in Gyeonggi-do and disaster managers from the Gyeonggi Disaster Support Group of the NEMC cooperatively developed C-MCIREM training for 6 months from March 1, 2019, to August 31, 2019 (Appendices/Supplement list and Appendices/Figure 5) [24].

C-MCIREM is based on knowledge education and tabletop map drill exercises reflecting the concepts of zone setup, chemical triage, decontamination, PPE, and antidote usage applying the chain of chemical survival proposed by Barelli et al with a training period of 7-8 hours in 1 day (Appendices/Table 3) [19]. Tabletop map drill exercises were designed to carry out training according to the scenario of a chemical disaster assigned with a virtual patient card on the map (Appendices/Supplement list). To enable objective evaluation of the map training practice in which trainees participate as a team and provide feedback for improvement, we searched and reviewed the literature within the last 15 years from PubMed, Google Scholar, and EMBASE using the keywords incident command system (ICS), hospital incident command system (HICS), disaster management, disaster mitigation, disaster preparedness, disaster response, disaster resilience, and chemical, biological, radiological, nuclear, and high yield explosives (CBRNE) disaster management.

After discussions with instructors, an evaluation index for tabletop map training was developed about chemical MCI or disaster (Appendices/Tables 3, 4) [2, 9, 19-23, 25-46]. In the knowledge and tabletop map training education, the concept of “pre-decontamination triage” proposed by Anan et al. was used as a chemical triage to prioritize decontamination of patients exposed to chemical substances in the field or hospital and reflected in the evaluation index of map training (Appendices/Tables 4, 5 and Appendices/Figure 4) [18-20]. In addition, we reflected chemical triage in the index of tabletop training in which the “proposed chemical mass casualty triage system” proposed by Cone et al. and the concept of “validating signs and symptoms of irritant gas syndrome agent (IGSA) exposure” proposed by Culley et al. were merged and applied to overcome the shortcomings of the START triage, which is the existing trauma-based disaster classification at the prehospital or hospital stage, and to determine the appropriate treatment for severe patients and the priority of antidote provision (Appendices/Tables 4, 5 and Appendices/Figure 4) [21, 23]. Finally, to evaluate the effectiveness of C-MCIREM and contribute to the improvement of education quality through future updates, we developed a survey for assessment of overall educational satisfaction.

Pilot implementation of C-MCIREM

Study Design, Selection of Participants, and Study Period

To confirm increases in knowledge, confidence, and competency in practical terms of disaster response personnel after C-MCIREM training, a pilot simulation study with a before and after the design was conducted (Appendices/Figures 3, 4 and Appendices/Figures 6-8). Participants' inclusion criteria were determined as those who voluntarily agreed to the training participation request among representative qualified paramedics who actually have worked at emergency medical service field, administrators of public health centers, and DMATs those who were assigned a role of deploying to the field according to the relevant national disaster law in Gyeonggi-do. The number of participants was determined based on the minimum number of participants for existing KDLS-Field and KDLS-Hospital education tabletop map training simulations (11 for the prehospital phase and 14 for the hospital phase) [17, 18]. On September 17, 2019, a total of 25 participants were recruited from fire departments (n = 5), public health centers (n = 4), and hospitals (n = 16) in Gyeonggi-do, South Korea, and received 7 hours of training in 1 day according to the C-MCIREM education timetable (Appendices/Table 3 and Appendices/Table 6).

Data Collection

All data in this study were collected and analyzed retrospectively from the pilot study of C-MCIREM education that had already been implemented. Data from all theory tests, confidence surveys, and satisfaction surveys were collected using an online Google survey with the consent of the participants. Knowledge tests and confidence surveys related to chemical MCI had a before and after design and results were collected online. In the tabletop map drill exercise, both quantitative and qualitative analyses were used to measure the educational effect on the teams made up of participants. There were 14 instructors consisting of the chief operating instructor who oversaw knowledge and tabletop map training, the tabletop training instructor, and 12 assistant training instructors (Appendices/Table 3). These 14 instructors were composed of disaster medical experts who completed both KDLS-Field and KDLS-Hospital, the national disaster education of the Republic of Korea, and were certified as disaster medical instructors in the country. They got acquainted with C-MCIREM programs beforehand by NEMC and researchers. The researchers set the ratio of instructor to the participant to be 2:1 in order to closely observe whether participants perform exactly what they need to do and to provide feedback. Of the 14, seven were disaster managers at NEMC, and seven were medical staff at hospitals. In addition, as those who participated in the production of C-MCIREM, they operated C-MCIREM before this pilot training and were trained in their role as instructors under the coordination of the chief operating instructor. In addition, the chief operating instructor was an emergency medicine (EM) specialist doctor at the hospital and a person who has authorized as an educational member of the KDLS education operational council under NEMC which empowered for his authority in the C-MCIREM production. In the tabletop map drill, all 14 instructors acted as rating staff in each allocated sector to evaluate and comment on the performance of team training using quantitative and qualitative methods, according to the tabletop map drill evaluation sheets for the prehospital team and hospital team (Appendices/Tables 3-5). The confidence survey related to chemical MCI and the survey for overall education assessment used an 11-point Likert scale, with a score of 0 being least favorable, 5 being neutral, and 10 being most favorable. This survey was conducted along with pre- and post-knowledge tests in the same format before and data were collected through Google survey.

Flowchart of C-MCIREM Training

**Figure 1:**
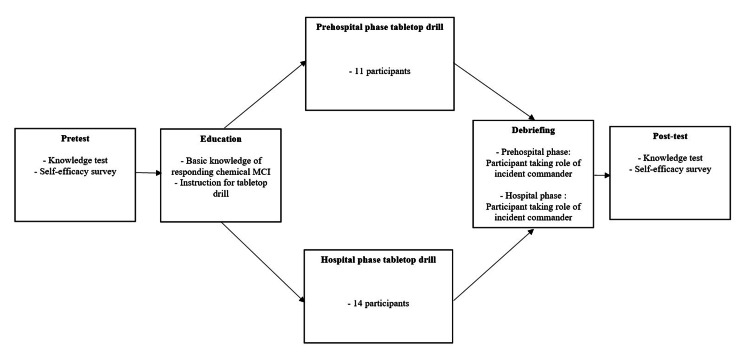
Flowchart of C-MCIREM Training Prehospital and hospital phase tabletop map drills proceed at the same time and be closely assessed by 14 instructors. C-MCIREM, Chemical-Mass Casualty Incident Response Education Module

The overall process of C-MCIREM training was summarized as a flowchart (Figure 1).

Scenario for the Tabletop Map Drill

In the tabletop map drill scenario, a large amount of hydrofluoric acid (HF) has leaked due to an explosion caused by a fire at a cell phone manufacturing plant in Bucheon, Gyeonggi-do, South Korea. At least 20 patients exposed to on-site hydrofluoric acid have been reported to the fire department emergency situation room.

Distribution of the Total of 25 Participants and Rule of Operation in the Tabletop Map Drill

Both the prehospital team and the hospital team, consisting of a total of 25 people, received a total of 25 minutes of training time for tabletop map drills after the opening of the scenario. Initially, there were seven participants in the prehospital team training area and 18 participants in the hospital team. After opening the scenario, the chief operating instructor informed the hospital team that four participants should be dispatched to the prehospital team as a hospital DMAT. Therefore, 11 personnel in the prehospital team and 14 in the hospital team were trained. After opening the scenario, the tabletop map training instructor inserted a total of 20 virtual patient cards with various exposure severities to HF at intervals into the prehospital team. The chief operating instructor increased hospital surges by inserting 20 triaged as minimal virtual patient cards that were not assigned to the prehospital team to the hospital team. The hospital team proceeded with treatment when the virtual patient card transferred from the prehospital team arrived at the hospital. The results of the survey on knowledge, confidence, and satisfaction for individuals were kept private to the trainees in the field, but the results of the quantitative evaluation of the tabletop map drill as teamwork were released after a debriefing of the participant taking the role of the incident commander and hospital incident commander of each team at the end of each session in the field, along with the comments of the rating staff.

Measurement Method of Knowledge Test Results

**Figure 2:**
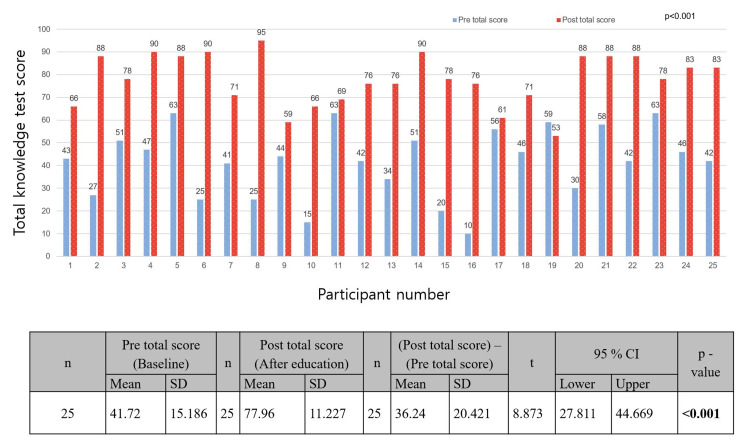
Comparison of pre- and post-training total scores of knowledge tests n, number; 95% CI, 95% confidence interval; Pre, pre-training; Post, post-training; SD, standard deviation. Statistical analyses were performed using the paired t-test.

The knowledge test consisted of three categories: disaster administration, disaster medicine, and preparedness and response to the chemical disaster. Baseline knowledge was measured before C-MCIREM training and a test was conducted after theory training to measure the improvement in knowledge (Table 1, Figure 2).

**Table 1:** Comparison of characteristics among institutions ADM, administration; EMT, emergency medical technician; IQR, interquartile range from 25% to 75%; n, number. Data are reported as the median (IQR) for continuous variables and number (%) for categorical variables. Statistical analyses were performed using Fisher’s exact test for categorical variables and the Kruskal-Wallis test for continuous variables.

Variable	Measure	Total (n = 25)	Fire Department (n = 5)	Public health center (n = 4)	Hospital (n = 16)	p-value
Age	median (IQR)	35 (28, 38)	31 (27, 40)	27 (25.75, 30.5)	35 (29.75, 38.25)	0.2613
Sex	n (%)	25 (100%)	5(100%)	4(100%)	16(100%)	0.362
Female		11 (44%)	1 (20%)	3 (75%)	7 (43.8%)	
Male		14 (56%)	4 (80%)	1 (25%)	9 (56.2%)	
Career (year)	median (IQR)	6 (2, 11)	6 (2, 6)	1 (1, 1.25)	10 (5.75, 11.25)	0.0054
Profession	n (%)	25 (100.0%)	5(100%)	4(100%)	16(100%)	0.109
EMT		3 (12.0%)	2 (40.0%)	0 (0.0%)	1 (6.2%)	
Nurse		9 (36.0%)	0 (0.0%)	3 (75.0%)	6 (37.5%)	
ADM		9 (36.0%)	3 (60.0%)	1 (25.0%)	5 (31.2%)	
Doctor		4 (16.0%)	0 (0.0%)	0 (0.0%)	4 (25.0%)	
Motivation for participation in training	n (%)	25 (100%)	5(100%)	4(100%)	16(100%)	0.0472
To prepare for upcoming national medical institution evaluation		9 (36.0%)	1 (20.0%)	2 (50.0%)	6 (37.50%)	
For one’s own interest		3 (12.0%)	3 (60.0%)	0	0	
At the recommendation of others		5 (20.0%)	0	0	5 (31.25%)	
No response		6 (24.0%)	1 (20.0%)	2 (50.0%)	3 (18.75%)	
Multiple (> 2)		2 (8.0%)	0	0	2 (12.50%)	
Pre-training total score of theoretical tests	median (IQR)	43 (30, 51)	51 (34, 51)	42.5 (41.75, 44)	43 (26.5, 56.75)	0.9835
Post-training total score of theoretical tests	median (IQR)	78 (71, 88)	78 (76, 88)	79.5 (69.75, 88.5)	78 (68.25, 88)	0.7553

Comparison of Characteristics of the 25 Participants

**Figure 3:**
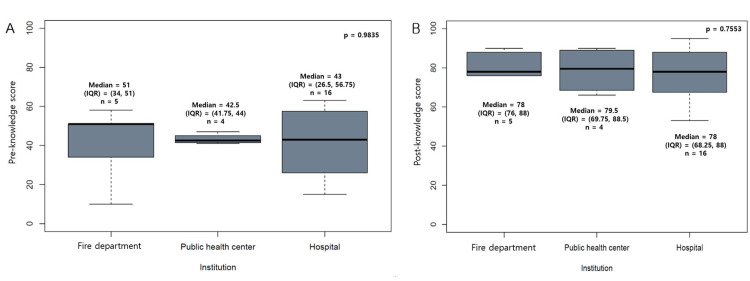
Comparison of pre- and post-training total scores of knowledge tests among institutions A: Comparison of the median (IQR) of pre-training total scores among professions (Left figure). B: Comparison of the median (IQR) of post-training test scores among professions (Right figure). IQR, interquartile range from 25% to 75%; Pre, pre-training; Post, post-training; Statistical analyses were performed using the Kruskal-Wallis test.

**Figure 4:**
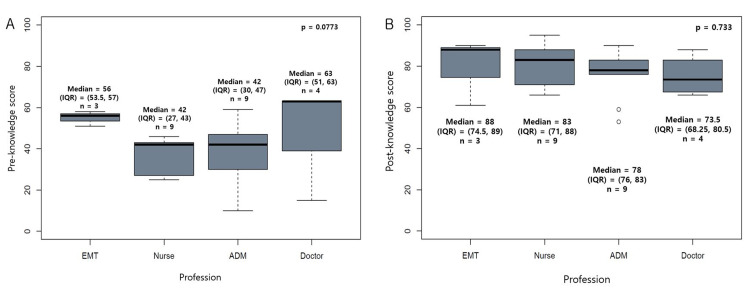
Comparison of the pre- and post-training total scores of knowledge tests among professions A: Comparison of the median (IQR) of pre-training total scores among professions (Left figure). B: Comparison of the median (IQR) of post test scores among professions (Right figure). ADM, administration; EMT, emergency medical technician; IQR, interquartile range from 25% to 75%; Pre, pre-training; Post, post-training; Statistical analyses were performed using the Kruskal-Wallis test.

**Figure 5:**
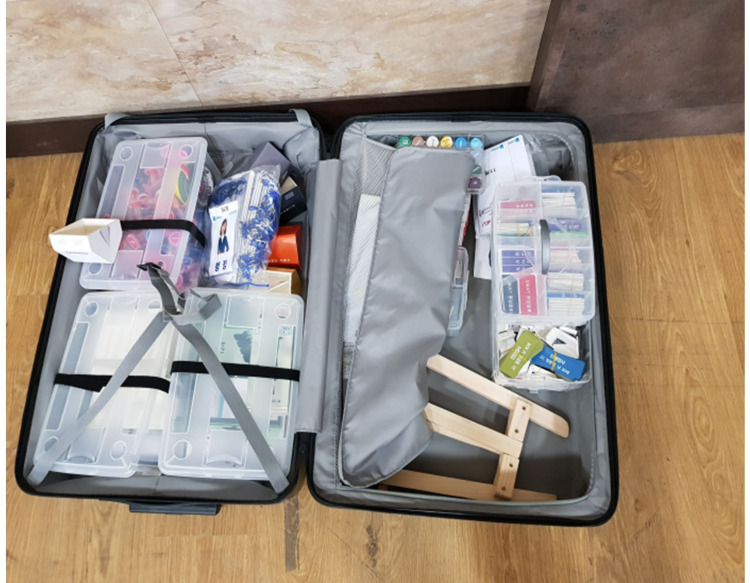
The tabletop drill exercise kit of C-MCIREM C-MCIREM, chemical-mass casualty incident response education module; the authors used this image on the courtesy of Gyeonggi Emergency Medical Support Center and Dawon Community Design (e-mail: ; Homepage: https://krscikr.modoo.at).

**Figure 6:**
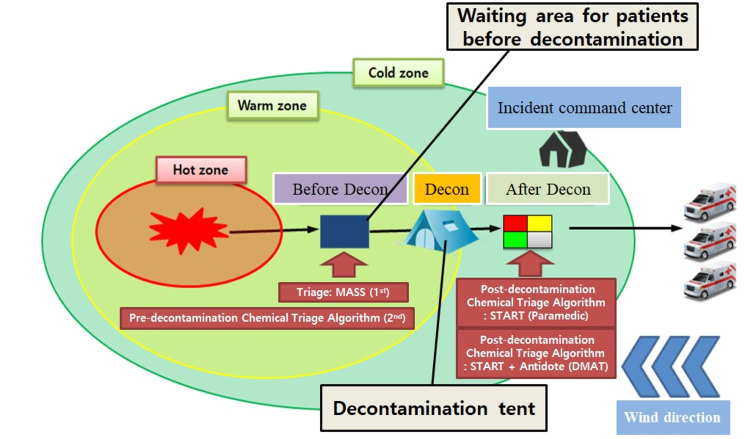
Triage concept in prehospital chemical MCI or disaster in the C-MCIREM C-MCIREM, chemical-mass casualty incident response education module; DMAT, disaster medical assistance team; MCI, multiple casualty incident; Decon, decontamination; The authors developed this “triage concept in prehospital chemical MCI or disaster in the C-MCIREM” by modifying previous basic chemical triage concept from the “DMAT's on-site response principle in the event of a chemical disaster” in emergency response manual of disaster emergency medical service (2016) in Republic of Korea [17, 18].

**Figure 7:**
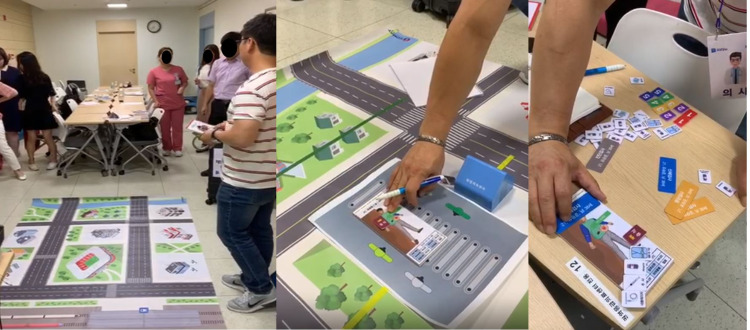
Development of C-MCIREM as prototypal version C-MCIREM, chemical-mass casualty incident response education module; the authors used this image on the courtesy of Gyeonggi Emergency Medical Support Center and Dawon Community Design (e-mail: ; homepage: https//krscikr.modoo.at).

**Figure 8:**
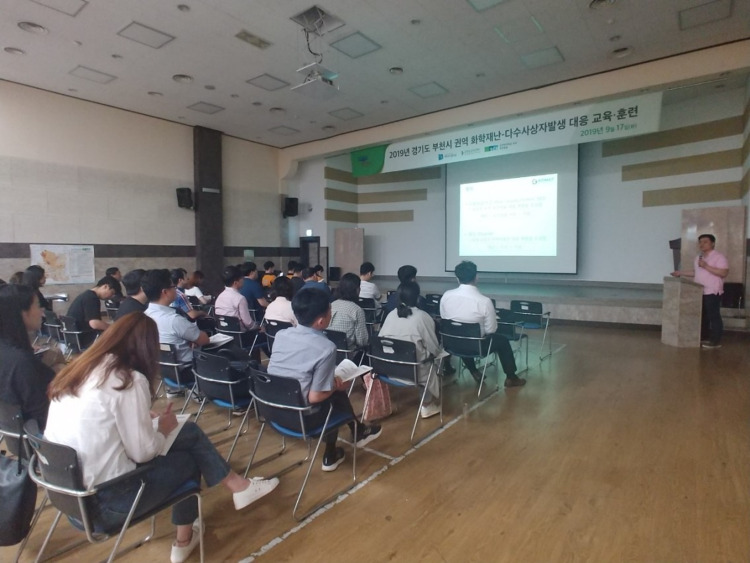
Knowledge education by chief operating instructor in C-MCIREM C-MCIREM, chemical-mass casualty incident response education module

The information variables of the total of 25 participants were age, sex, career (years), profession, and motivation for participation in the training (Appendices/Table 6). The characteristics of the total of 25 participants were compared according to the institution and profession, including the pre- and post-training total knowledge test scores (Tables 1, 2, Figures 3, 4).

**Table 2:** Comparison of characteristics according to the profession ADM, administration; EMT, emergency medical technician; IQR, interquartile range from 25% to 75%; n, number. Data are reported as the median (IQR) for continuous variables and number (%) for categorical variables. Statistical analyses were performed using Fisher’s exact test for categorical variables and the Kruskal-Wallis test for continuous variables.

Variable	Measure	Total (n = 25)	EMT (n = 3)	Nurse (n = 9)	ADM (n = 9)	Doctor (n = 4)	p-value
Age	median (IQR)	35 (28, 38)	28 (27.5, 29.5)	30 (26, 35)	39 (29, 40)	37.5 (36.5, 38)	0.0833
Sex	n (%)	25 (100%)	3 (100%)	9(100%)	9(100%)	4(100%)	0.015
Female		11 (44%)	2 (66.7%)	7 (77.8%)	1 (11.1%)	1 (25%)	
Male		14 (56%)	1 (33.3%)	2 (22.2%)	8 (88.9%)	3 (75%)	
Career (years)	median (IQR)	6 (2, 11)	6 (6, 6)	5 (1, 9)	7 (2, 11)	10.5 (10, 11.25)	0.1809
Institution	n (%)	25 (100.0%)	3 (100%)	9(100%)	9(100%)	4(100%)	0.109
Fire department		5 (20.0%)	2 (66.7%)	0 (0.0%)	3 (33.33%)	0 (0.0%)	
Public health center		4 (16.0%)	0 (0.0%)	3 (33.33%)	1 (11.1%)	0 (0.0%)	
Hospital		16 (64.0%)	1 (33.3%)	6 (66.7%)	5 (55.6%)	4 (100.0%)	
Motivation for participation in training	n (%)	25 (100%)	3 (100%)	9(100%)	9(100%)	4(100%)	0.4559
To prepare for upcoming national medical institution evaluation		9 (36.0%)	0	3 (33.33%)	4 (44.44%)	2 (50.0%)	
For one’s own interest		3 (12.0%)	2 (66.67%)	0	1 (11.11%)	0	
At the recommendation of others		5 (20.0%)	1 (33.33%)	2 (22.22%)	2 (22.22%)	0	
No response		6 (24.0%)	0	3 (33.33%)	2 (22.22%)	1 (25.0%)	
Multiple (> 2)		2 (8.0%)	0	1 (11.11%)	0	1 (25.0%)	
Pre-training total score of theoretical tests	median (IQR)	43 (30, 51)	56 (53.5, 57)	42 (27, 43)	42 (30, 47)	63 (51, 63)	0.0773
Post-training total score of theoretical tests	median (IQR)	78 (71, 88)	88 (74.5, 89)	83 (71, 88)	78 (76, 83)	73.5 (68.25, 80.5)	0.733

Measurement Method of Confidence Survey Score Results for Chemical Multiple Casualty Incident Responses

The confidence survey scores obtained using an 11-point Likert scale for multiple casualty chemical incident response in the total of 25 participants were compared between baseline and after training (Table 3).

**Table 3:** Comparison of pre- and post-training confidence survey score results for chemical multiple casualty incident responses IQR, interquartile range from 25% to 75%; n, number; MCI, multiple casualty incident; PPE, personal protective equipment; SD, standard deviation; Likert scale, 11-point Likert scale with a score of 0 being lowest confidence, 5 being neutral, and 10 being highest confidence. Statistical analyses were performed using the paired t-test or Wilcoxon’s signed rank-sum test for continuous variables.

Confidence survey questions for chemical MCI response	n	Pre-training Likert scores (Baseline)		Min/Max	Pre-training Likert scores (Baseline)		n	Post-training Likert scores		Min/Max	Post-training Likert scores		p-value
		Median	IQR		Mean	SD		Median	IQR		Mean	SD	
Appropriate chemical MCI response	25	3	2-5	0/10	3.52	2.40	25	7	5.5-8	5/10	7.08	1.68	
PPE selection and usage	25	3	2-5	0/10	3.68	2.58	25	7	6-8.5	5/10	7.20	1.66	
Appropriate antidote selection	25	2	0.5-5	0/8	2.92	2.41	25	7	5.5-8	3/10	7.04	1.84	
Antidote stockpiling and passing on knowhow to colleagues	25	3	0-5	0/8	2.64	2.22	25	7	6.5-9	2/10	7.32	1.99	
Zone setup and decontamination	25	3	0.5-5	0/7	2.84	2.19	25	7	7-9	5/10	7.60	1.63	
Chemical triage before and after decontamination	25	3	0-5	0/9	2.72	2.56	25	7	7-9	5/10	7.64	1.66	

Measurement Method of Tabletop Map Drill Test Results

After the demonstration by the instructor, the participants were divided into a prehospital team and a hospital team and performed a total of two tabletop map drills. According to the tabletop map drill evaluation sheets for prehospital and hospital teams in C-MCIREM, the results evaluated by a total of 14 instructors have scored pre- and post-training, and compared using a quantitative method (Tables 4, 5).

**Table 4:** Tabletop drill results for the prehospital team DMAT, disaster medical assistance team; ICS, incident command system; PPE, personal protective equipment, EMT, emergency medical technician; Sandglass timer is introduced in the Supplement list. The authors developed this evaluation sheet after a review of the literature about concepts of disaster management including special chemical disaster situations [2, 9, 19-23, 25-46].

Assessment category	Evaluation target	Evaluation items satisfied at first drill	Earned Score at first drill	Evaluation items satisfied at second drill	Earned score at second drill	Total score per category	Detailed evaluation items (score)	Rater number (Comment)
1. Early on-site disaster declaration	On-site EMT	a	2	a, b	4	4	a. Declaration of a disaster situation “This is a disaster site” (2)	Rater 1
							b. Situation control “Please follow my instructions” (2)	
2. Activation of disaster response system	On-site EMT	c, d	4	a, b, c, d	8	8	a. Patient location (2)	
							b. Expected number of patients (2)	
							c. Type of disaster (2)	
							d. Transition to a disaster response system (2)	
3. On-site initial Mass Triage	On-site EMT		0		0	4	a. Identification and gathering of people who can walk (2)	
							b. Identification of conscious patients among patients who are unable to walk (2)	
4. On-site zone and facility setup	ICS commander	b, c	4	a, b, c	6	6	a. Setup of the incident command center, temporary on-site emergency medical facility, temporary on-site morgue, press center, and communication network (2)	
							b. Zone setup to assist PPE level and decontamination decision making (2)	
							c. Decontamination tent installation considering wind direction (2)	
5. Casualty status board setup	ICS commander		0	b	2	4	a. Instruction to install casualty status board (2)	
							b. Instruct team members to report to the ICS commander in real-time (2)	
6. Use of regional chemical hazard surveillance map	ICS commander	a	2	a	2	2	a. Determining the PPE level to wear (2)	Rater 2 (In the first drill, some of the members of the DMAT team went into the decontamination tent without wearing PPE.)
7. Decontamination	ICS commander	a, c, d	6	a, c, d, e	8	12	a. Instructing and supervising pre-decontamination triage algorithm (2)	Rater 3
							b. Instructing and supervising decontamination of patients (2)	
							c. Instructing decontamination team members to undress the patients (2)	
							d. Instructing and supervising post-decontamination triage algorithm (2)	
							e. Instructing team members to provide new clothing to patients who have completed decontamination (2)	
							f. Instructing team members to clean up waste after removing PPE and to decontaminate themselves (2)	
8. Triage	Triage team	a = 0 (case) b = 0 (case)	20	a = 0 (case) b = 0 (case)	20	20	Principle 1. Patient triage method (1st: mass triage; 2nd: pre-decontamination triage; 3rd: post-decontamination triage (START) as a sequence) (deducted from a total of 20 points). 2. If patient triage is not performed, the total score is 0. 3. If the ratio of the triaged patients to all patients put into the drill is	Rater 4 (In the first and second drills, all 20 patients put into the drill were triaged appropriately as 7 immediate, 3 urgent, 7 minimal, and 3 deceased by the START method.)
							a. Under-triage (−2 per case)	
							For example: immediate (red) - > urgent (yellow) or urgent (yellow) - > minimal (green) or immediate (red) - > minimal (green)	
							b. Over-triage (−1 per case)	
							For example: minimal (green) - > urgent (yellow) or urgent (yellow)- > immediate (red) or minimal (green) - > immediate (red) or deceased (black) - > immediate (red)	
9. Management of patients triaged as minimal	Triage team		0		0	2	a. Explain waiting to patients triaged as minimal in the designated district and restrict movement (2)	Rater 2
10. Transport to hospital	Transport team	c	18	a = 2 (case)	16	20	Hospital selection according to triage (deducted from a total of 20 points)	Rater 5 (In the first drill, seven patients triaged as minimal were transported to one hospital at a time.)
							Principle: 1. All patients triaged as immediate should be transported to the hospital with level 1 or 2 emergency medical center within the surge limit; 2. All patients triaged as urgent or minimal should be transported to the hospital within the surge limit.	
							a. Improper transport to hospital for patients triaged as immediate (−2 per case)	
							b. Failure of transport to a hospital with proper distribution for patients triaged as urgent (−2)	
							c. Failure of transport to a hospital with proper distribution for patients triaged as minimal (−2)	
11. Response to the media	ICS commander	a	2	a, b	4	4	a. Confirmation of setup of press center (2)	Rater 1
							b. ICS commander check whether the press center setup has been done or not. ICS commander puts the media on hold in a designated location that does not interfere with field activities until an official briefing is conducted if press center setup has not been done (2)	
12. Re-triage	DMAT leader	a, b	4	a, b	4	4	a. Instructing re-triage for patients triaged as minimal (2)	Rater 2
							b. Evaluating accuracy of re-triage (2)	
13. Debriefing to the media	ICS commander	c	2	a, b, c, d, e	10	10	a. Debriefing including patient location (2)	Rater 1
							b. Debriefing including expected number of patients (2)	
							c. Debriefing including the type of disaster (2)	
							d. Debriefing including casualty scale (2)	
							e. Debriefing including the status of transport to hospital for the total patient population (2)	
14. Time relevance	All participants	α = 0	0	α = 0	0	α	The termination criterion is when the transport of all patients and preparation of the status board are completed. Overtime is calculated as 1 point per minute after the designated 25 minutes of the drill and is deducted from the total score. α = overtime	Tabletop training instructor as Rater 13
Total score	The team score and overall proficiency	Baseline	64	Improved	84	100 - α	Total score = Sum of scores from categories 1 to 13 − α	The chief operating instructor as Rater 14

**Table 5:** Tabletop drill results of the hospital team CT, computed tomography; DMAT, disaster medical assistance team; ED, emergency department; Hb, hemoglobin; HICS, hospital incident command system; ICU, intensive care unit; OPD, outpatient department; PPE, personal protective equipment.; Sandglass timer was introduced in the Supplement. The authors developed this evaluation sheet after a review of the literature about concepts of disaster management including special chemical disaster situations [2, 9, 19-23, 25-46].

Assessment category	Evaluation target	Evaluation items satisfied at first drill	Earned Score at first drill	Evaluation items satisfied at second drill	Earned score at second drill	Total score per category	Detailed evaluation items (score)	Rater number (Comment)
1. Early hospital disaster declaration	ED director	a, b	2	a, b	2	2	a. Immediate recognition and activation of HICS (1)	Rater 6
							b. Transition to the hospital disaster response system by phone call to the HICS commander (1)	
2. Declaration of transition to hospital disaster system	HICS commander	e, g	2	a, b, e, f, g	5	7	a. Mention of expected number of patients (1)	
							b. Mention of transition to disaster care to secure disaster medical resources (1)	
							c. Activation of the internal hospital emergency contact network (1)	
							d. Activation of the external hospital emergency contact network (1)	
							e. Check whether the DMAT is dispatched from the hospital (1)	
							f. Secure in-hospital communication system (e.g., social network service) (1)	
							g. Setup of hospital disaster response headquarters (1)	
3. Use of regional chemical hazard surveillance map and antidote preparedness	ED director	a, c, d	3	a, b	2	4	a. External hospital zone setup as hot, warm, and cold for decontamination (1)	Rater 7
							b. Decontamination tent setup considering the wind direction (1)	
							c. Determining the PPE level to wear by the regional chemical hazard surveillance map (1)	
							d. Instructions for hospital preparedness of antidote by the regional chemical hazard surveillance map (1)	
4. Decontamination	ED director		0	a, b, c	6	12	a. Instruction and supervision of pre-decontamination triage algorithm (2)	
							b. Instruction and supervision of decontamination of patients (2)	
							c. Instruction of decontamination team members to remove the patients’ existing clothes (2)	
							d. Instruction and supervision of post-decontamination triage algorithm (2)	
							e. Instruction of team members to provide new clothing to patients who have completed decontamination (2)	
							f. Instruction of team members to clean up waste after removing PPE and to decontaminate themselves (2)	
5. Initial patient zoning in ED	All participants	a = 3 (case)	0	a = 0 (case)	6	6	Principle: Initial patient zone allocation should be accomplished according to the severity (deducted from a total of 6 points)	Rater 8
							a. Transport of a minor severity patient to ICU zone (−2 per case and maximum −6)	
6. Expansion of ED and setup of HICS facility	HICS commander	d	2	a, b, c, d, e	10	10	a. Setup of triage area outside the ED (2)	Rater 9
							b. Setup of guardian waiting area outside the ED (2)	
							c. Setup of patient area triaged as minimal outside the ED (2)	
							d. Setup of hospital command center (2)	
							e. Setup of press center (2)	
7. Enhancing security	HICS commander		0		0	2	a. Establishment of limit line on entrance and exit of the hospital including the ED (1)	Rater 10
							b. Placement of security personnel at each entrance (1)	
8. Securing ED beds	ED director	A	2	a, b, c	6	6	a. Early disposition, such as hospitalization or discharge of patients not related to the disaster in the ED (2)	
							b. Zone rearrangement of stabilized patients among disaster-related patients in the ED (2)	
							c. Instruction of relatively stable patients among immediate patients moving to the ED warded bed in case of ED ICU bed lacking (2)	
9. Securing disaster reserve beds and available hospital facilities	HICS commander		0	b, c	4	6	a. Instruction to open disaster reserve beds (2)	Rater 10
							b. Instruction to open extra outpatient CT room (2)	
							c. Instruction to open extra outpatient X-ray room (2)	
10. Manpower reinforcement in the ED	HICS commander		0	a, b, c, d	8	8	a. Doctor (2)	Rater 9
							b. Nurse (2)	
							c. Patient transfer staff (2)	
							d. Security personnel (2)	
11. Securing available resources	HICS commander	a, b, c, d	8	c, d, e, f, g, h	12	20	a. Instruction to secure ventilator (2)	Rater 11 (At the first drill, the medical staff in charge of the non-emergency zone should ask the HICS commander to reinforce personnel, to resolve patient congestion. In addition, the medical staff in charge of the emergency zone should quickly identify patients who have finished surgery and admit them to the ICU.)
							b. Instruction to secure blood products (2)	
							c. Instruction to secure ICU beds (2)	
							d. Instruction to secure operating rooms (2)	
							e. Instruction to secure human resources from the general surgery department (2)	
							f. Instruction to secure human resources from the neurosurgery department (2)	
							g. Instruction to secure human resources from thoracic surgery department (2)	
							h. Instruction to secure human resources from orthopedic surgery department (2)	
							i. Instruction to secure human resources from the anesthesiology department (2)	
							j. Instruction to secure human resources from pharmacy department (2)	
12. Re-triage	ED director	a	0	a	0	4	Instructing re-triage by emergency medical center director a. No instruction = 0 b. Instruction once only = 2 c. Instruction more than 2 times = 4	Rater 8
13. Time constraint on the action in treating severe patients	All participants	b		a, b, d		No allocated score in this category but delays to the drilling process by sandglass timer of a 1- or 2-minute span per item	a. Applying a 1-minute sandglass timer on central line catheterization or chest tube insertion, respectively	Rater 8
							b. Applying each 1-minute sandglass timer if the imaging tests or diagnostic tests per patient are performed	Rater 10
							c. Applying a 2-minute sandglass timer if there are cardiac arrest events or seizure attack events, respectively,	Rater 8
							d. Applying a 2-minute sandglass timer if there are operations or interventions, respectively,	Rater 11
							f. Applying a 1-minute sandglass timer on the omission of inpatient or outpatient referral confirmation signature	Rater 10
14. Operation of bottleneck areas	HICS commander	a	1	a, b, c	3	3	a. Instruction to increase reception staff more than twice (1)	Rater 12
							b. Instruction of reception staff to dispatch to the parking lot to receive numerous patients outside the hospital (1)	
							c. Instruction of dispatch of reception staff to receive patients who first moved to each zone within the EM (1)	
	ED director	a = 0 (case)	6	a = 0 (case)	6	6	In principle, all suspected fracture patients should be X-rayed (deducted from a total of 6 points)	Rater 10
							a. Number of waiting patients in the imaging room exceeds 3 (−2 per case and maximum −6)	
15. Debriefing to the media	HICS commander	b	1	a, b, c	3	4	a. Debriefing including the type of disaster (1)	Rater 6
							b. Debriefing including casualty scale (1)	
							c. Debriefing including the number of patients undergoing surgery and ICU admission (1)	
							d. Debriefing including the transition to hospital disaster system - surge control in OPD and ED as well as secured hospitalization beds (1)	
16. Appropriateness of treatment and resource utilization	All participants	h = 1 (case)	−1			Whenever there is a corresponding item, the total score is deducted.	a. No surgery for patients in need (−5)	Rater 12 (At the first drill, the number of patients was incorrectly checked as having 1 more than the original in the debriefing.)
							b. No intervention for patients in need (−5)	
							c. No thoracotomy for patients in need (−3)	
							d. No transfusion to patients with Hb 7 or lower (−3)	
							e. No more than intravenous line hydration in case of hypotension (−3)	
							f. No intubation for patients in need (−3)	
							g. No use after opening the disaster reserve bed (−3)	
							h. Error of patient counting (−1 per case)	
Total score	The team score and overall proficiency	Baseline	26	Improved	73	100	Total score = Sum of scores from categories 1 to 16	The chief operating instructor as Rater 14

Measurement Method of Survey for Overall Education Assessment

After finishing the tabletop map training exercise, a survey of overall education assessment was implemented online and the results were analyzed quantitatively (Table 6).

**Table 6:** Survey results for overall educational satisfaction assessment in 22 participants n, number; Min, minimum; Max, maximum; SD, standard deviation. An 11-point Likert scale was used for the survey with a score of 0 being least favorable, 5 being neutral, and 10 being most favorable.

Survey item	n	Score (Min)	Score (Max)	Mean	SD
1. Willingness to recommend this training program to others	22	2	10	8.18	1.893
2. Overall satisfaction score for the theoretical education	22	2	10	8.64	1.733
3. Overall satisfaction score for tabletop training	22	2	10	8.41	1.869
4. Beneficial and effective in terms of training skills and knowledge transfer	22	6	10	8.73	1.162
5. Thinking this education is necessary for professional health workers	22	7	10	8.82	1.097
6. Policymakers should also receive this training program	22	6	10	8.91	1.192

Statistical Analysis

Data were reported as the mean and standard deviation (SD) in the parametric test or the median and interquartile range (IQR) from 25% to 75% (Q1/Q3) with minimum to maximum (Min/Max) in the non-parametric test for continuous variables and number (%) for categorical variables. Continuous variables were analyzed using the Kruskal-Wallis test and categorical variables were analyzed using Fisher’s exact test. In all analyses, p < 0.05 was taken to indicate statistical significance. Statistical analyses were performed using Rex Excel-based statistical analysis software (version 3.0.3; RexSoft Inc., Seoul, South Korea).

## Results

Knowledge test results

A total of 25 (100%) participants, 14 men (56%) and 11 women (44%), took the pre- and post-training knowledge tests (Table 1, Figure 2). The median (Q1, Q3) of age and number of career years of the total of 25 participants were 35 (28, 38) years and 6 (2. 11) years, respectively (Tables 1, 2). In the knowledge test, the mean (SD) scores at baseline and after education for all 25 participants were 41.72 (15.19) and 77.96 (11.23), respectively (*p* < 0.001) (Figure 2). The mean (SD) difference between the total pre- and post-training knowledge test scores was 36.24 (20.42) (t = 8.873, 95% CI = 27.81-44.67; *p* < 0.001) (Figure 2).

Comparing characteristics of the total of 25 participants

The characteristics of the 25 participants were compared according to institution and profession (Tables 1, 2). In the comparison by institution, the median (IQR) of career was 1 (1-1.25) year in public health centers and 10 (5.75-11.25) years in hospitals (p = 0.0054). Pre- and post-training total scores of knowledge tests showed no differences according to the type of institution (*p* = 0.9835 and *p* = 0.7553, respectively) (Figure 3) or profession (*p* = 0.0773 and *p* = 0.733, respectively (Figure 4, Tables 1, 2).

Confidence survey score results for chemical multiple casualty incident responses

In the chemical MCI response survey of all 25 participants, the median (IQR) scores with Min/Max regarding confidence at baseline and after training were increased for appropriate chemical MCI response [3 (2-5), 0/10 vs. 7 (5.5-8), 5/10, respectively; *p* < 0.001], PPE selection and usage [3 (2-5), 0/10 vs. 7 (6-8.5), 5/10, respectively; *p* < 0.001], appropriate antidote selection [2 (0.5-5, 0/8 vs. 7 (5.5-8), 3/10, respectively; *p* < 0.001], antidote stockpiling and passing on knowhow to colleagues [3 (0-5), 0/8 vs. 7 (6.5-9), 2/10, respectively; *p* < 0.001], zone setup and decontamination [3 (0.5-5), 0/7 vs. 7 (7-9), 2/10, respectively; *p* < 0.001], and chemical triage before and after decontamination [3 (0-5), 0/9 vs. 7 (7-9), 5/10, respectively; *p* < 0.001] (Table 3).

Tabletop map drill test results

Prehospital Team

In the quantitative measurement of qualitative analysis of tabletop map training drill simulation in the prehospital team composed of 11 participants, the total scores in the pre- and post-training drills were 64/100 and 84/100, respectively (Table 4). On qualitative analysis, the prehospital team showed improvements after training in Early on-site disaster declaration; Activation of disaster response system; On-site zone and facility setup; Casualty status board setup; Decontamination; Response to the media; Debriefing to the media; and Team score and overall proficiency (Table 4). The prehospital team had satisfactory assessments on both pre- and post-training drills for Use of regional chemical hazard surveillance map; Triage; Re-triage; and Time relevance (Table 4). However, there were no improvements following training for On-site initial mass triage; or Management of patients triaged as minimal. In addition, the score for Transport to the hospital was worsened in the post-training drill with a value of −4 due to two cases of improper transport to the hospital for patients triaged as immediate (−2 per case) compared to the value of −2 in the pre-training drill due to failure of transport to hospital for patients triaged as minimal (−2), where seven patients triaged as minimal were transported to one hospital at a time (Table 4).

Hospital Team

In the quantitative measurement of qualitative analysis of tabletop map training drill simulation in the hospital team composed of 14 participants, the total scores in the pre- and post-training drills were 26/100 and 73/100, respectively (Table 5). On qualitative analysis of pre- and post-training drills, the hospital team showed improvements for: Declaration of transition to hospital disaster system; Decontamination; Initial patient zoning in ED; Expansion of ED and setup of HICS facility; Securing ED beds; Securing disaster reserve beds and available hospital facilities; Manpower reinforcement in the ED; Securing available resources; Time constraint action in treating severe patients; Operation of bottleneck areas by HICS commander; Debriefing to the media; Appropriateness of treatment and resource utilization; and Team score and overall proficiency. The hospital team had satisfactory assessments on both pre- and post-training drills for Early hospital disaster declaration; and the Operation of bottleneck areas by the ED director (Table 5). However, there was no improvement after training for Enhancing security; or Re-triage (Table 5). Moreover, the post-training assessment decreased for: Use of regional chemical hazard surveillance map and antidote preparedness with a value of 2 satisfying items of zone setup and decontamination tent setup considering the wind direction compared to the pre-training drill with a value of 3 satisfying items of zone setup, determining the PPE level to wear by the regional chemical hazard surveillance map, and instructions for hospital preparedness of antidote by the regional chemical hazard surveillance map (Table 5). After pre- and post-training exercise debriefings, Rater 11 commented that the medical staff in charge of the non-emergency zone should ask the HICS commander to reinforce personnel, to resolve patient congestion and that the medical staff in charge of the emergency zone should quickly identify patients who have finished surgery and admit them to the ICU (Table 5).

Survey results for overall education assessment

Twenty-two participants out of the total 25 (88%) responded to the survey regarding the overall assessment of the training program and showed at least over 8 out of 10 in the mean value (Table 6).

## Discussion

This pilot training with the C-MCIREM achieved an increase in knowledge for a total of 25 participants, increased confidence in preparing and responding to chemical MCI, and increased disaster medical response capabilities through tabletop map drill disaster exercises, as determined by quantitative and qualitative methodologies. In particular, in the case of the knowledge test, there was no gap between groups according to characteristics or organization, which confirmed that this training program was delivered evenly and effectively. In the final questionnaire, the participants gave the training program a high satisfaction score. As a pilot study, this training program had only 25 participants (11 for the prehospital phase and 14 for the hospital phase), the minimally required number for KDLS-Field and KDLS-Hospital training programs. Future studies with increased numbers of participants are expected to provide a more objective evaluation of the effectiveness of the training program. In particular, it is not possible to judge statistical significance with a single training session. Conducting C-MCIREM training at least three times would allow comparison of the quantitative scores for each team and, therefore, the educational effect of tabletop map training would be amenable to statistical analysis.

In this C-MCIREM pilot training program, post-decontamination chemical triage at the prehospital stage was divided between DMATs and EMTs (Appendices/Figure 4). This was because of the limitation of the special legal scope of the job among Korean EMTs [47]. According to the Emergency Response Manual in Disaster Fields published by the Ministry of Health and Welfare in 2016, the DMAT team adopts the Sort, Assess, Life-saving interventions, Treatment and/or transport (SALT) multi-injury severity classification method as the basis for patient classification, but the START method can also be used in combination for individual patients [17, 18]. On the other hand, EMTs cannot apply SALT, which requires life-saving intervention, because the scope of their work is limited according to the Korean Medical Service Act and Emergency Medical Service Act. Therefore, in this training program, only START triage was adopted at the prehospital stage after decontamination [17, 18, 47].

EMTs, who account for the largest proportion of emergency personnel who are the first to encounter patients in a hazardous chemical exposure accident, can provide no treatment other than simple trauma treatment and only classify and transfer patients. Therefore, patients are unable to receive life-saving antidote treatment until arrival at the hospital. This was reflected in this C-MCIREM training program (Appendices/Figure 4). Prehospital Disaster medical response practitioners and participants in this C-MCIREM pilot training program answered question #6 (Policymakers should also receive this training program) with a mean (SD) score of 8.91 (1.192). This reflects the opinion of field operatives that such legislative obstacles should be improved. Under the premise that they have appropriate knowledge and skills to respond to each hazardous chemical and a regular evaluation system is in place, adjusting the scope of work for EMTs to permit active treatment in the field through regular medical guidance would have a positive effect on the prognosis of patients. Such legal and institutional differences can be adjusted according to local circumstances in different countries, and so they will not have a significant impact on the universal applicability of C-MCIREM training programs, but such differences in national and regional systems and laws should not be overlooked.

A chemical hazard surveillance map applying the concept of hazard vulnerability assessment was produced and used in the simulation for prehospital and hospital disaster preparedness and response (Appendices/Supplement list). Using this map, with local data of hazardous chemical materials, disaster response health care personnel can set up zones, determine decontamination methods, determine PPE levels, and make decisions regarding antidote preparation and use. Although this chemical hazard surveillance map was created based on regional data from Bucheon, a city in Gyeonggi-do, South Korea, its scope can be expanded to other countries, which is a major advantage of this C-MCIREM map training method.

In addition to the MASS triage/START Triage, which is the existing trauma-based disaster classification, the participants newly experienced the chemical disaster response triage presented in the past disaster medical literature [20, 21, 23]. Thus, in this training assuming chemical MCI, participants set up the zone, selected the appropriate PPE and decontamination method and priority, and learned how to use antidote correctly. The authors evaluate this as a major achievement of the C-MCIREM program.

However, global social distancing and restrictions on group members due to the 2020 COVID-19 pandemic became obstacles to the continued implementation of this C-MCIREM training program with the existing platform. In the future, theoretical training should be converted to an online platform, and the number of participants should be limited so that instructors and trainees can participate in the tabletop map drill exercise only after COVID-19 vaccination, where offline training is indispensable. To ensure effective and safe education, it is necessary to develop operational plans that take into account reality, such as converting to the original education format after the achievement of herd immunity.

This study had some limitations in that it was a one-off pilot study. The sample size of the total participants was small at 25. However, in tabletop map simulation training, after the instructor’s demonstration, the well-organized curriculum, scoring system, and feedback system through a total of two repetitions maximized the advantages of indirect education, such as simulation, with low cost and high efficiency. The authors expect that, as more training is provided in the future, the degree of completion of training itself will increase. Indeed, the authors are proceeding with the future study to compare the result of this pilot study to the result of the study which will meet the sample size criteria after multiple education completion.

## Conclusions

In this study, the C-MCIREM education program created with the support of Gyeonggi-do, the National Municipality of South Korea, was implemented as a pilot trial. In addition to the existing MASS/START disaster triage, pre-decontamination triage and post-decontamination chemical triage were reflected in knowledge and tabletop map drill training. In addition, zone setup, PPE selection, antidote selection, and basic supportive care were also reflected in the overall education for chemical disaster response. The C-MCIREM including chemical disaster tabletop map drill exercise had clear educational benefits, both individually and as a team, and clearly strengthened the disaster preparedness confidence of the participants including the overall satisfaction of the education. In order to prove the universal educational effect of this C-MCIREM in the future, more education is needed.

## Figures and Tables

**Figure 9:**
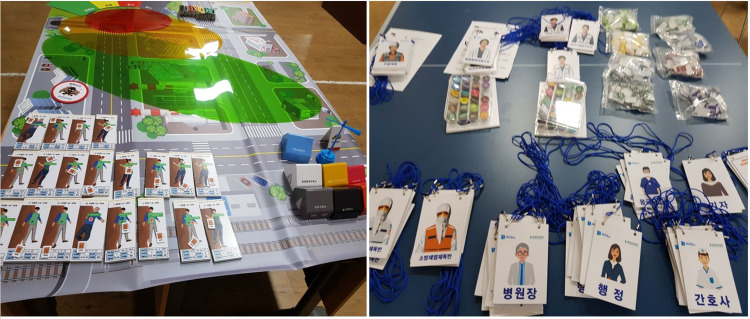
Prehospital setting for tabletop map drill exercise in C-MCIREM Left: Prehospital map, patient card, zone setup, triage tags, and other instruments to play tabletop exercise; Right: Role card necklace and management interaction magnetics for participants; C-MCIREM, chemical-mass casualty incident response education module; the authors used this image on the courtesy of Gyeonggi Emergency Medical Support Center and Dawon Community Design (e-mail: ; homepage: https//krscikr.modoo.at).

**Figure 10:**
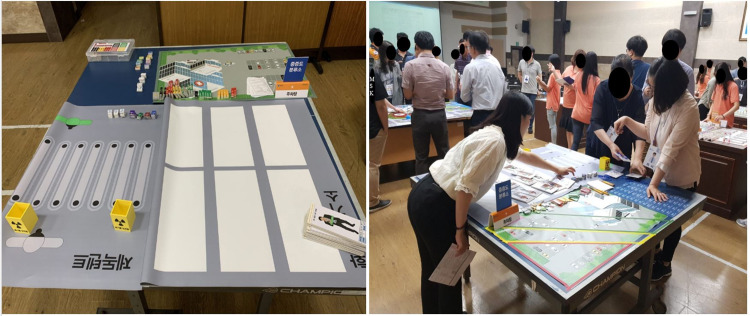
Hospital setting for tabletop map drill exercise in C-MCIREM Left: Hospital map, hospital parking lot as triage sector, waiting area for patients before decontamination, decontamination tent, patient card, triage tags, and other instruments to play tabletop exercise; Right: Operating map table in hospital setting for tabletop drill exercise; C-MCIREM, chemical-mass casualty incident response education module; the authors used this image on the courtesy of Gyeonggi Emergency Medical Support Center and Dawon Community Design (e-mail: ; homepage: https//krscikr.modoo.at).

**Figure 11:**
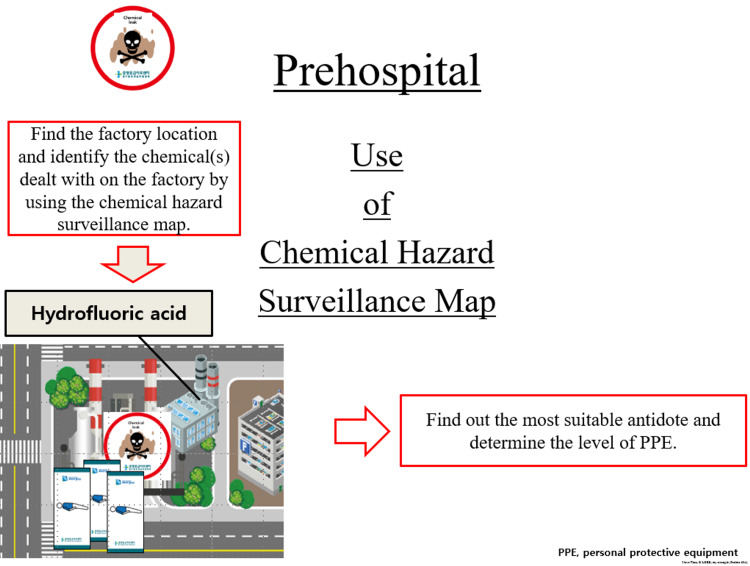
Prehospital use of chemical hazard surveillance map in the C-MCIREM C-MCIREM, chemical-mass casualty incident response education module; the authors developed this “prehospital use of chemical hazard surveillance map in the C-MCIREM”.

**Figure 12:**
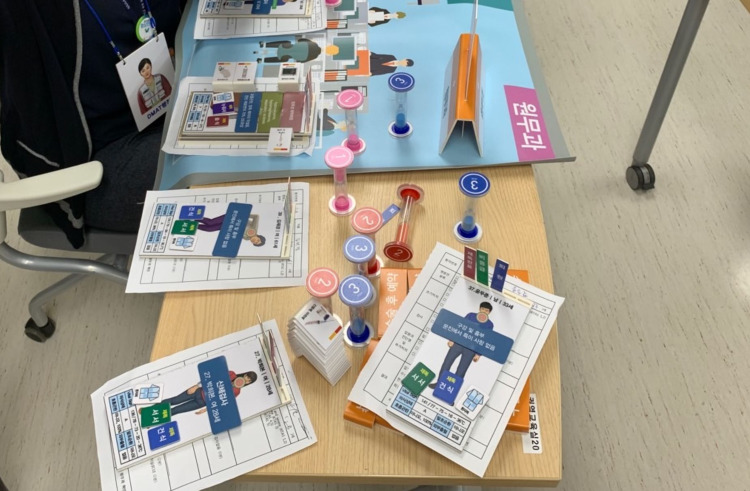
Examples of 1-, 2-, and 3-minute sandglass timers used in the C-MCIREM

## Appendices

**Table 7:** Timetable for C-MCIREM training C-MCIREM, Chemical-Mass Casualty Incident Response Education Module; HICS, hospital incident command system; ICS, incident command system; MCI, mass casualty incident.

Time	Title	Presenter or moderator (n) and remarks if necessary
09:00–09:30	Operational meeting before C-MCIREM education for the chief operating instructor, tabletop training instructor, and 12 assistant tabletop training instructors	Chief operating instructor (1) Tabletop training instructor (1) Assistant training instructors (12) All 14 instructors performed the role of 14 rater staff simultaneously in each allocated sector to evaluate and comment on the performance of team training using quantitative and qualitative methods.
09:30–10:00	Registration of trainees including pre-training knowledge test and pre-training confidence level survey (20 min) for chemical MCI response	Chief operating instructor (1)
10:00–11:00	1. Lecture on disaster response system in South Korea 2. Lecture on prehospital and hospital disaster medical responses	Chief operating instructor (1)
11:00–11:10	Break	
11:10–12:00	1. Introduction of regional chemical hazard surveillance map 2. Lecture on steps for chemical MCI response, including brief video clip explaining the tabletop drill simulation	Chief operating instructor (1)
12:00–13:00	Lunch break	
13:00–13:30	Lecture on tabletop training for prehospital and hospital chemical MCI response	Tabletop training instructor (1)
13:30–14:20	1. Detailed explanation of tabletop training kit and exercise demonstration by all instructors (30 min) 2. Debriefing of each incident commander from prehospital and hospital teams for chemical MCI response (10 min) 3. Questions and answers (10 min)	Chief operating instructor (1) Tabletop training instructor (1) Assistant training instructors (12)
14:20–14:30	Break	
14:30–15:15	1. First tabletop drill as prehospital and hospital teams for chemical MCI response by the total of 25 participants (25 min) 2. Five-minute debriefings of both ICS and HICS commanders for chemical MCI response (10 min) 3. Feedback comments by all instructors and assessors (10 min)	Chief operating instructor (1) Tabletop training instructor (1) Assistant training instructors (12) All 14 instructors performed roles of 14 rater staff simultaneously in each allocated sector to evaluate and comment on the performance of team training using quantitative and qualitative methods.
15:15–15:25	Break	
15:25–15:55	1. Post - training knowledge test and post-training confidence level survey for chemical MCI response (20 min)	
15:55–16:40	1. Second tabletop drill as prehospital and hospital teams for chemical MCI response by the total of 25 participants (25 min) 2. Debriefing of each incident commander from prehospital and hospital teams for chemical MCI response (10 min) 3. Feedback comments by all instructors and assessor staff (10 min)	Chief operating instructor (1) Tabletop training instructor (1) Assistant training instructors (12) All 14 instructors performed the roles of 14 rater staff simultaneously in each allocated sector to evaluate and comment on the performance of team training using quantitative and qualitative methods.
16:40–17:00	1. Survey for overall educational satisfaction assessment (10 min) 2. Take home messages, questions, and answers	

**Table 8:** Evaluation sheet of tabletop drill for the prehospital team in C-MCIREM DMAT, disaster medical assistance team; ICS, incident command system; PPE, personal protective equipment; The authors developed this evaluation sheet after the review of literatures about concepts of disaster management including special chemical disaster situations [2, 9, 19–23, 25–46].

Assessment category	Evaluation target	Evaluation items satisfied at first drill	Earned Score at first drill	Evaluation items satisfied at second drill	Earned score at second drill	Total score per category	Detailed evaluation items (score)	Rater number (Comment)
1. Early on-site disaster declaration	On-site EMT					4	a. Declaration of a disaster situation “This is a disaster site” (2)	Rater 1
							b. Situation control “Please follow my instructions” (2)	
2. Activation of disaster response system	On-site EMT					8	a. Patient location (2)	
							b. Expected number of patients (2)	
							c. Type of disaster (2)	
							d. Transition to a disaster response system (2)	
3. On-site initial Mass Triage	On-site EMT					4	a. Identification and gathering of people who can walk (2)	
							b. Identification of conscious patients among patients who are unable to walk (2)	
4. On-site zone and facility setup	ICS commander					6	a. Setup of incident command center, temporary on-site emergency medical facility, temporary on-site morgue, press center, and communication network (2)	
							b. Zone setup to assist PPE level and decontamination decision making (2)	
							c. Decontamination tent installation considering wind direction (2)	
5. Casualty status board setup	ICS commander					4	a. Instruction to install casualty status board (2)	
							b. Instruct team members to report to ICS commander in real time (2)	
6. Use of regional chemical hazard surveillance map	ICS commander					2	a. Determining the PPE level to wear (2)	Rater 2
7. Decontamination	ICS commander					12	a. Instructing and supervising pre-decontamination triage algorithm (2)	Rater 3
							b. Instructing and supervising decontamination of patients (2)	
							c. Instructing decontamination team members to undress the patients (2)	
							d. Instructing and supervising post-decontamination triage algorithm (2)	
							e. Instructing team members to provide new clothing to patients who have completed decontamination (2)	
							f. Instructing team members to clean up waste after removing PPE and to decontaminate themselves (2)	
8. Triage	Triage team					20	Principle 1. Patient triage method (1st: mass triage; 2nd: pre-decontamination triage; 3rd: post-decontamination triage (START) as sequence) (deducted from a total of 20 points). 2. If patient triage is not performed, the total score is 0. 3. If the ratio of the triaged patients to all patients put into the drill is	Rater 4
							a. Under-triage (−2 per case)	
							For example: immediate (red) - > urgent (yellow) or urgent (yellow) - > minimal (green) or immediate (red) - > minimal (green)	
							b. Over-triage (−1 per case)	
							For example: minimal (green) - > urgent (yellow) or urgent (yellow)- > immediate (red) or minimal (green) - > immediate (red) or deceased (black) - > immediate (red)	
9. Management of patients triaged as minimal	Triage team					2	a. Explain waiting to patients triaged as minimal in designated district and restrict movement (2)	Rater 2
10. Transport to hospital	Transport team					20	Hospital selection according to triage (deducted from a total of 20 points)	Rater 5
							Principle: 1. All patients triaged as immediate should be transported to hospital with level 1 or 2 emergency medical center within the surge limit; 2. All patients triaged as urgent or minimal should be transported to hospital within the surge limit.	
							a. Improper transport to hospital for patients triaged as immediate (−2 per case)	
							b. Failure of transport to hospital with proper distribution for patients triaged as urgent (−2)	
							c. Failure of transport to hospital with proper distribution for patients triaged as minimal (−2)	
11. Response to the media	ICS commander					4	a. Confirmation of setup of press center (2)	Rater 1
							b. ICS commander check whether press center setup has been done or not. ICS commander puts the media on hold in a designated location that does not interfere with field activities until an official briefing is conducted if press center setup has not been done (2)	
12. Re-triage	DMAT leader					4	a. Instructing re-triage for patients triaged as minimal (2)	Rater 2
							b. Evaluating accuracy of re-triage (2)	
13. Debriefing to the media	ICS commander					10	a. Debriefing including patient location (2)	Rater 1
							b. Debriefing including expected number of patients (2)	
							c. Debriefing including type of disaster (2)	
							d. Debriefing including casualty scale (2)	
							e. Debriefing including status of transport to hospital for the total patient population (2)	
14. Time relevance	All participants					α	The termination criterion is when the transport of all patients and preparation of the status board are completed. Overtime is calculated as 1 point per minute after the designated 25 minutes of the drill and is deducted from the total score. α = overtime	Tabletop training instructor as Rater 13
Total score	Team score and overall proficiency	Baseline		Comment		100 - α	Total score = Sum of scores from categories 1 to 13 − α	Chief operating instructor as Rater 14

**Table 9:** Evaluation sheet of tabletop drill for hospital team in C-MCIREM CT, computed tomography; DMAT, disaster medical assistance team; ED, emergency department; Hb, hemoglobin; HICS, hospital incident command system; ICU, intensive care unit; OPD, outpatient department; PPE, personal protective equipment; The authors developed this evaluation sheet after the review of literatures about concepts of disaster management including special chemical disaster situation. 2, 9, 19–23, 25–46

Assessment category	Evaluation target	Evaluation items satisfied at first drill	Earned Score at first drill	Evaluation items satisfied at second drill	Earned score at second drill	Total score per category	Detailed evaluation items (score)	Rater number (Comment)
1. Early hospital disaster declaration	ED director					2	a. Immediate recognition and activation of HICS (1)	Rater 6
							b. Transition to the hospital disaster response system by phone call to the HICS commander (1)	
2. Declaration of transition to hospital disaster system	HICS commander					7	a. Mention of expected number of patients (1)	
							b. Mention of transition to disaster care to secure disaster medical resources (1)	
							c. Activation of the internal hospital emergency contact network (1)	
							d. Activation of the external hospital emergency contact network (1)	
							e. Check whether the DMAT is dispatched from the hospital (1)	
							f. Secure in-hospital communication system (e.g., social network service) (1)	
							g. Setup of hospital disaster response headquarters (1)	
3. Use of regional chemical hazard surveillance map and antidote preparedness	ED director					4	a. External hospital zone setup as hot, warm, and cold for decontamination (1)	Rater 7
							b. Decontamination tent setup considering the wind direction (1)	
							c. Determining the PPE level to wear by the regional chemical hazard surveillance map (1)	
							d. Instructions for hospital preparedness of antidote by the regional chemical hazard surveillance map (1)	
4. Decontamination	ED director					12	a. Instruction and supervision of pre-decontamination triage algorithm (2)	
							b. Instruction and supervision of decontamination of patients (2)	
							c. Instruction of decontamination team members to remove the patients’ existing clothes (2)	
							d. Instruction and supervision of post-decontamination triage algorithm (2)	
							e. Instruction of team members to provide new clothing to patients who have completed decontamination (2)	
							f. Instruction of team members to clean up waste after removing PPE and to decontaminate themselves (2)	
5. Initial patient zoning in ED	All participants					6	Principle: Initial patient zone allocation should be accomplished according to the severity (deducted from a total of 6 points)	Rater 8
							a. Transport of a minor severity patient to ICU zone (−2 per case and maximum −6)	
6. Expansion of ED and setup of HICS facility	HICS commander					10	a. Setup of triage area outside the ED (2)	Rater 9
							b. Setup of guardian waiting area outside the ED (2)	
							c. Setup of patient area triaged as minimal outside the ED (2)	
							d. Setup of hospital command center (2)	
							e. Setup of press center (2)	
7. Enhancing security	HICS commander					2	a. Establishment of limit line on entrance and exit of the hospital including the ED (1)	Rater 10
							b. Placement of security personnel at each entrance (1)	
8. Securing ED beds	ED director					6	a. Early disposition, such as hospitalization or discharge of patients not related to disaster in the ED (2)	
							b. Zone rearrangement of stabilized patients among disaster-related patients in the ED (2)	
							c. Instruction of relatively stable patients among immediate patients moving to the ED warded bed in case of ED ICU bed lacking (2)	
9. Securing disaster reserve beds and available hospital facilities	HICS commander					6	a. Instruction to open disaster reserve beds (2)	Rater 10
							b. Instruction to open extra outpatient CT room (2)	
							c. Instruction to open extra outpatient X-ray room (2)	
10. Manpower reinforcement in the ED	HICS commander					8	a. Doctor (2)	Rater 9
							b. Nurse (2)	
							c. Patient transfer staff (2)	
							d. Security personnel (2)	
11. Securing available resources	HICS commander					20	a. Instruction to secure ventilator (2)	Rater 11
							b. Instruction to secure blood products (2)	
							c. Instruction to secure ICU beds (2)	
							d. Instruction to secure operating rooms (2)	
							e. Instruction to secure human resources from general surgery department (2)	
							f. Instruction to secure human resources from neurosurgery department (2)	
							g. Instruction to secure human resources from thoracic surgery department (2)	
							h. Instruction to secure human resources from orthopedic surgery department (2)	
							i. Instruction to secure human resources from anesthesiology department (2)	
							j. Instruction to secure human resources from pharmacy department (2)	
12. Re-triage	ED director					4	Instructing re-triage by emergency medical center director a. No instruction = 0 b. Instruction once only = 2 c. Instruction more than 2 times = 4	Rater 8
13. Time constraint on action in treating severe patients	All participants					No allocated score in this category but delays to the drill process by sandglass timer of a 1- or 2-minute span per item	a. Applying 1-minute sandglass timer on central line catheterization or chest tube insertion, respectively	Rater 8
							b. Applying each 1-minute sandglass timer if the imaging tests or diagnostic tests per patient are performed	Rater 10
							c. Applying 2-minute sandglass timer if there are cardiac arrest events or seizure attack events, respectively,	Rater 8
							d. Applying 2-minute sandglass timer if there are operations or interventions, respectively,	Rater 11
							f. Applying 1-minute sandglass timer on the omission of inpatient or outpatient referral confirmation signature	Rater 10
14. Operation of bottleneck areas	HICS commander					3	a. Instruction to increase reception staff more than twice (1)	Rater 12
							b. Instruction of reception staff to dispatch to the parking lot to receive numerous patients outside the hospital (1)	
							c. Instruction of dispatch of reception staff to receive patients who first moved to each zone within the EM (1)	
	ED director					6	In principle, all suspected fracture patients should be X-rayed (deducted from a total of 6 points)	Rater 10
							a. Number of waiting patients in the imaging room exceeds 3 (−2 per case and maximum −6)	
15. Debriefing to the media	HICS commander					4	a. Debriefing including type of disaster (1)	Rater 6
							b. Debriefing including casualty scale (1)	
							c. Debriefing including number of patients undergoing surgery and ICU admission (1)	
							d. Debriefing including transition to hospital disaster system - surge control in OPD and ED as well as secured hospitalization beds (1)	
16. Appropriateness of treatment and resource utilization	All participants					Whenever there is a corresponding item, the total score is deducted.	a. No surgery for patients in need (−5)	Rater 12
							b. No intervention for patients in need (−5)	
							c. No thoracotomy for patients in need (−3)	
							d. No transfusion to patients with Hb 7 or lower (−3)	
							e. No more than intravenous line hydration in case of hypotension (−3)	
							f. No intubation for patients in need (−3)	
							g. No use after opening the disaster reserve bed (−3)	
							h. Error of patient counting (−1 per case)	
Total score	Team score and overall proficiency	Baseline		Comment		100	Total score = Sum of scores from categories 1 to 16	Chief operating instructor as Rater 14

Supplement list

1. Development of C-MCIREM as prototypal version (Figure 5)

2. Supplementary Table 1. Basic information of total 25 participants (Table 6)

**Table 10:** Basic information of total of 25 participants ADM, administration; M, male; F, female; EMT, emergency medical technician

Trainee number	Gender	Age	Institution	Career (year)	Licensure	Motivation of education participation (multiple answer possible)
1	F	25	Public health center	1	Nurse	To prepare for upcoming national medical institution evaluation
2	M	27	Hospital	1	Nurse	By the recommendation of others
3	M	40	Firehouse	7	ADM	For one's own interest
4	M	28	Public health center	2	ADM	To prepare for upcoming national medical institution evaluation
5	M	38	Hospital	12	Doctor	To prepare for upcoming national medical institution evaluation
6	M	25	Hospital	5	Nurse	To prepare for upcoming national medical institution evaluation
7	F	26	Public health center	1	Nurse	No reply
8	F	39	Hospital	14	Nurse	No reply
9	M	35	Hospital	11	ADM	To prepare for upcoming national medical institution evaluation
10	M	35	Hospital	10	Doctor	To prepare for upcoming national medical institution evaluation
11	M	37	Hospital	10	Doctor	To prepare for upcoming national medical institution evaluation, by the recommendation of others
12	F	31	Hospital	9	Nurse	To prepare for upcoming national medical institution evaluation, by the recommendation of others
13	M	53	Firehouse	1	ADM	To prepare for upcoming national medical institution evaluation
14	F	31	Firehouse	6	Paramedic (EMT)	For one's own interest
15	M	40	Hospital	11	ADM	By the recommendation of others
16	M	27	Firehouse	2	ADM	No reply
17	F	28	Hospital	6	EMT	By the recommendation of others
18	F	35	Hospital	10	Nurse	To prepare for upcoming national medical institution evaluation
19	M	39	Hospital	14	ADM	No reply
20	F	52	Hospital	24	ADM	To prepare for upcoming national medical institution evaluation
21	M	27	Firehouse	6	Paramedic (EMT)	For one's own interest
22	F	38	Public health center	1	Nurse	No reply
23	F	38	Hospital	11	Doctor	No reply
24	F	30	Hospital	5	Nurse	By the recommendation of others
25	M	29	Hospital	2	ADM	By the recommendation of others

3. Supplementary Figure 1. Knowledge education by chief operating instructor in C-MCIREM (Figure 6)

4. Supplementary Figure 2. Prehospital setting for tabletop map drill exercise in C-MCIREM (Figure 7)

5. Supplementary Figure 3. Hospital setting for tabletop map drill exercise in C-MCIREM (Figure 8)

6. Prehospital use of chemical hazard surveillance map in the C-MCIREM (Figure 9)

7. Example of 1-, 2-, and 3-minute sandglass timer used in the C-MCIREM (Figure 10)
